# Survey on the Developments of Unmanned Marine Vehicles: Intelligence and Cooperation

**DOI:** 10.3390/s23104643

**Published:** 2023-05-10

**Authors:** Inyeong Bae, Jungpyo Hong

**Affiliations:** Department of Information and Communication Engineering, Changwon National University, Changwon 51140, Republic of Korea; 20150053@changwon.ac.kr

**Keywords:** artificial intelligence, cooperation, swarm, unmanned marine vehicle

## Abstract

With the recent development of artificial intelligence (AI) and information and communication technology, manned vehicles operated by humans used on the ground, air, and sea are evolving into unmanned vehicles (UVs) that operate without human intervention. In particular, unmanned marine vehicles (UMVs), including unmanned underwater vehicles (UUVs) and unmanned surface vehicles (USVs), have the potential to complete maritime tasks that are unachievable for manned vehicles, lower the risk of man power, raise the power required to carry out military missions, and reap huge economic benefits. The aim of this review is to identify past and current trends in UMV development and present insights into future UMV development. The review discusses the potential benefits of UMVs, including completing maritime tasks that are unachievable for manned vehicles, lowering the risk of human intervention, and increasing power for military missions and economic benefits. However, the development of UMVs has been relatively tardy compared to that of UVs used on the ground and in the air due to adverse environments for UMV operation. This review highlights the challenges in developing UMVs, particularly in adverse environments, and the need for continued advancements in communication and networking technologies, navigation and sound exploration technologies, and multivehicle mission planning technologies to improve UMV cooperation and intelligence. Furthermore, the review identifies the importance of incorporating AI and machine learning technologies in UMVs to enhance their autonomy and ability to perform complex tasks. Overall, this review provides insights into the current state and future directions for UMV development.

## 1. Introduction

During World War II, many weapons incorporating state-of-the-art technology were developed [1], and unmanned marine vehicle (UMV) technology appeared for the first time in smoke screen operations to clear mines [2]. Additionally, UMVs were utilized to assess combat damage, collect water samples, and recover lost equipment [3]. Each nation tried to win during the war by keeping the details of its weaponry a secret. In particular, underwater surveillance technology was confidential until the end of the Cold War after which technologies for unmanned vehicles (UVs) began to develop in earnest.

UVs can operate in all environments including the air, ground, sea, and even underwater. They can keep up with battlefield conditions by gathering, analyzing, and validating data using sensors. In general, UVs can be used to safely explore without loss of life dangerous or undeveloped areas that cannot be easily accessed by humans because they are operated without a human aboard. In addition, disregarding the limitation of day and night, these can operate for an extended period; therefore, equipment expenses and mission input costs can be lower than those with manned vehicles.

Early UVs were controlled remotely by operators. However, they have now developed into advanced unmanned ground vehicles (UGVs), unmanned aerial vehicles (UAVs), and UMVs, which move autonomously by recognizing and judging the surrounding environment or inputting preprepared programs. These UVs can be used in various systems with combat platforms allied with other UVs [4,5].

Initially, the UGV was developed for the purpose of explosive ordnance disposal (EOD); mine clearance; and intelligence, surveillance, and reconnaissance (ISR). These days, the UGV has moved away from its initial military development goals and is expanding in its scope to supply and agriculture [6,7]. In addition, UAVs have the advantage of being able to control the movement of unmanned aircraft remotely, making it easy to scout and acquire information even in dangerous areas that are difficult for humans to access [8,9]. UAVs are suited for surveillance scenarios because they can explore patrol regions at a relatively high speed using cameras and can provide excellent communication capabilities [10]. Recently, UAVs have been utilized not only for military purposes [11] but also for civilian ones in various fields, such as shipping, aerial photography, agriculture, and weather observation [12,13].

Recently, UMVs have been highlighted as a key component of future naval operations in missions; that is, mine control, maritime security, and maritime blockade operations [11]. In general, UMVs include USVs and UUVs [1], and UUVs can be divided into remotely operated vehicles (ROVs), which are wired to surface ships, and autonomous underwater vehicles (AUVs), which are completely wireless. UUVs can be sent to deeper waters that are inaccessible to humans, using onboard cameras and sonar sensors to explore underwater, with a robot arm capable of taking samples [13]. UUVs are widely used for both military and civilian purposes, such as marine research, detecting and clearing mines, and long-range reconnaissance.

**Figure 1:**
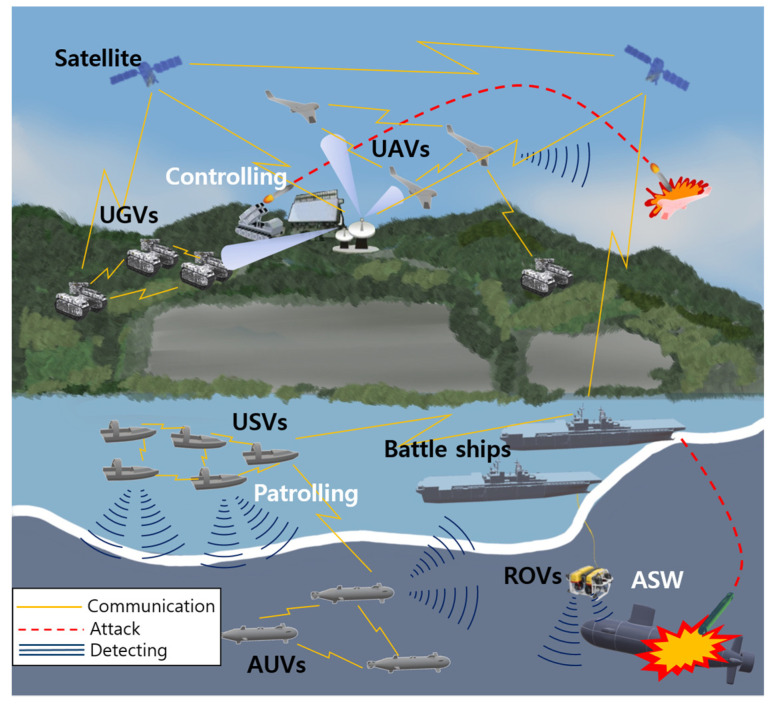
Concept of future battlefield operations.

A new heterogeneous swarm surveillance system that goes beyond a single platform and monitors the entire area in cooperation with UAVs, UGVs, USVs, and UUVs has been actively studied [5,14,15,16,17,18,19]. From a single platform, the fields of land, sea, air, space, electromagnetics, and networks are increasingly becoming interconnected in the battlespace, transforming into a multiplatform combat mode of multiple environments [20], as shown in Figure 1.

Multirobot systems were created to overcome the limitations of single robots, which include their lack of information-processing capability and inability to perform specialized tasks [21,22]. Since the concept was first introduced in the late 1980s, cooperative robots have been defined, and concepts such as cellular robotics, collective robotics, and distributed robotics have been proposed. The swarm unmanned system applies swarm intelligence, which consists of performing collective actions in natural ecosystems to robots so that multiple unmanned vehicles capable of exchanging information work together and operate homogeneously for the same purpose. Robots equipped with such swarm intelligence is referred to as ‘swarm robotics’ [4,5]. The data acquired by multiple unmanned swarm robots are shared and fused to perform joint tasks, accurately recognizing the surrounding situation more effectively. This includes control technology that avoids collisions with vehicles or obstacles that run close to each other while generating information by sharing and fusing the data acquired by each component as well as collaborating technology that shares tasks in real-time in response to changing environments. A new subarea of research, swarm robotics, has emerged to increase flexibility and fault tolerance through use of multiple robots, borrowing the shape of life in motion with synergistic packs [21]. Swarm robotic systems deal with complex systems in which entities interact with each other using minimal communication with neighboring entities to derive a single global creative action [22]. The swarmbot project was first introduced in the 2000s and is still being studied in various fields.

Recently, studies using cooperation between unmanned vehicles, such as UAV–UGV and UAV–USV combinations, have been actively conducted [23,24,25,26,27,28,29,30,31,32,33]. In the ocean, when a UUV and USV are used together rather than alone, they are more accurate and can carry out missions and reduce both operational time and cost [34]. In addition, there has been a significant trend toward the development of advanced sensors in unmanned maritime vehicles (UMVs). Recently, sensors have become lighter and miniaturized in size. For example, STMicroelectronics’ L3GD20H model is a gyro sensor that uses small-sized MEMS technology. It contains a three-axis gyroscope, which shrinks to 3mm × 3mm × 1 mm in size. In addition, lightweight sonar sensors in small UUVs include various models, such as the Ping Ultrasonic Distance Sensor, which usually range from a few millimeters to several tens of centimeters in size, usually less than 20 mm in length and width.

In summary, unmanned system technology is being developed both individually and cooperatively in various fields. This study focuses on UMVs and analyzes the most recent trends and future direction of UMV development to address the growing importance of unmanned systems in the ocean, such as mine countermeasure (MCM) and antisubmarine warfare (ASW), among other advances in different areas.

The reference materials selected in this paper were comprehensively selected from the basic data published in the past to the data that are currently being studied. Recently, for the rapid development of technology, private technologies have also been widely accepted as defense technologies, so data have been collected from a large body of civilian research in addition to that from the navy.

The paper begins by discussing past and modern UMVs in Section 2, which is followed by an introduction of essential sensors in Section 3. Proposed studies of UMV intelligence are presented in Section 4, while Section 5 introduces swarm and cooperation of UMVs in other fields. Finally, we provide a discussion and conclusion in Section 6 and Section 7, respectively.

## 2. History and Current Development Status of UMVs

### 2.1. Unmanned Surface Vehicles (USVs)

An USV is an unmanned marine system that navigates and performs missions using its own controls. USVs are relatively small and cost-effective, and are used in large marine surface areas. They are also being increasingly deployed in missions that require very long patrol times in dangerous areas because humans perform missions almost individually with little coordination [35,36,37,38]. In the case of USVs, several USV developments have been carried out through public and private planning for various fields and purposes [39,40], and USVs are being commercially used in scientific research, environmental missions, marine exploration, military applications, and other applications, such as traffic, communication relay, and fuel supply [41].

USVs first appeared during World War II, but it was not until the 1990s that their use was extended to carrying out national projects in earnest [42,43]. The first USV was the Comox, developed in 1944 in Canada [1]. Comox was developed for Operation Normandy to perform smoke screen operations on behalf of airplanes. It was responsible for supporting mine sweeping and the demolition of rocket craft; although the experiment was successful, it was not used in practice. Around the same time, the United States developed and demonstrated USVs namely ‘Porcupine’, ‘Bob-Sled’, and ‘Woofus 120′ equipped with mine-removing rockets to remove mines and obstacles in a region. Able and Baker began using drone boats to collect radiation samples after an atomic bomb detonation in 1946. A USV was developed and used as a target for missile launch training in the 1960s [43,44]. Even today, many target USVs, including seaborne-powered targets (SEPARs), high-speed maneuverable seaborne targets (HSMST), and mobile ship targets (MSTs), QST-33 and QST-35/35A [44], are still in service as unmanned ground ships. USVs began to be used for mining purposes after the 1950s. In the late 1960s, the larger minesweeping drone USV was also developed and deployed in Vietnam, where it was used for mine removal. By the 1990s, R/C Dyads, Moss, and an advanced lightweight influence sweep system (ALISS) were developed for demining; more recently, the sophisticated USV demining systems, namely Brown, Palmer, and Brizzola, were developed. It was only in the late 1990s that the USV began to be increasingly used for a variety of purposes, including information/monitoring/reconnaissance, port surveillance, and maritime surveys. Autonomous search and hydrographic vehicles (ASHs) and Roboski were initially developed as shipboard deployed surface targets (SDSTs); that is, as a jet ski-type target for ship self-defense training. They are currently used in difficult missions, such as reconnaissance [1,45].

#### 2.1.1. USV Type

The US Navy defined standards for efficiently developing USVs in 2007, called the USV Master Plan [46]. The USV Master Plan classifies USVs, which are limited to marine vessels, based on the USV craft type or size.

##### USV Craft Types

The classification and mission according to the craft types of the USV is shown in Table 1. 

##### USV Size

The classification according to the size of the USV is shown in Table 2, and the details are presented in [48,49]:

#### 2.1.2. Application

**Figure 2:**
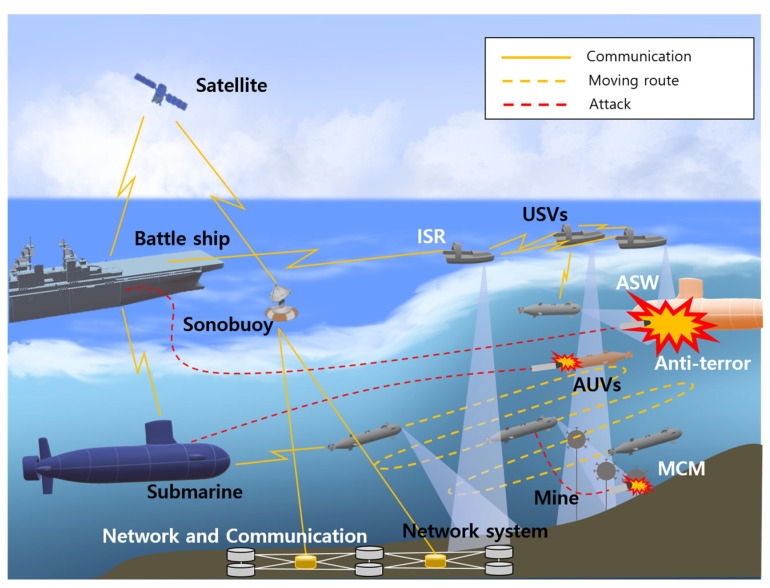
Schematic diagram of typical functions of USVs. Deployment of a USV–UUV formation is also possible.

The main applications of USVs are MCM, antiterror, ISR, ASW, and marine exploration [46,50]. The schematic diagram of USV applications is shown in Figure 2, and USV–UUV cooperation is briefly presented. Detailed explanations are as follows:

i. MCM

The unmanned platform can be operated without the need for manned platforms to enter areas suspected of having mines [51]. The fleet can be operated by finding or removing areas without mines, and the sailing schedule can be shortened. USVs are used by large fleets to establish safe operating areas, transport routes, and transport lanes quickly. Mines found using the USV are neutralized using the device’s onboard minesweeper. It is necessary to operate within a naval system quickly, independent of other combat capabilities.

ii. Antiterror

Integrated into a broader security network, the USV provides port and riverside security against stealth and nontraditional threats [46]. The USV performs day and night port or river monitoring remotely over an extended period. If it is determined during an investigation of a potential threat platform that the platform poses a threat, the USV is in charge of removing it. It also provides remote detection, interrogation, and engagement with potential threats to port surveillance, merchant ships, and naval vessels.

iii. ISR

Monitoring should be possible to perform successfully at sea or on the coast in accordance with the general instructions of commanders and operators and regardless of the danger of the situation [52]. The USV is equipped with a forward-oriented infrared laser distance meter used to detect and track nearby targets and an on-mount camera that can operate day and night. A constant link between the USV and control station should be maintained for monitoring via the USV.

ISR capabilities can help identify suspicious behaviors in maritime traffic, eliminate hazards, and protect marine facilities and ships. In addition, the mission completed by the USV will provide enhanced capability and flexibility to operational maritime units because command and control center personnel can operate from a distance.

iv. ASW

Effective deployment and maintenance of ASW capabilities against increasing submarine threats are critical [53]. ASWs operate using the relative positions of sensors and snipers, and a variety of variations—single-state (transmitters and receivers deployed on a single USV), dual-state, and multistate approaches (transmitters and receivers deployed on various platforms/USVs). This allows the USV to complement and extend the existing ASW capabilities using specific USVs based on other available assets and capabilities.

In addition, USVs can efficiently perform tasks on behalf of humans in dangerous situations, such as maritime rescue and disaster prevention pilot support, marine territorial protection, marine environment exploration, and torpedo operation support.

v. Marine exploration

Ocean exploration is mainly performed by research teams or private companies using USVs. The Woods Hole Oceanographic Institution (WHOI) in the United States has developed Mesobot [54] for tracking mesopelagic zone organisms. It is about the size of a travel suitcase (1.5 m long, 1.5 m high, 1 m wide) and was developed to track organisms in the mesopelagic zone or twilight-zone. Mesobot is also equipped with an additional ultrasonic-based fish detection and tracking system to track the light-averse, deep-sea organisms that inhabit the mesopelagic zone. The USV ocean exploration field has been pioneered by the United States. In particular, the wave adaptive modular vessel (WAM-V) [55], an unmanned surface ship developed by Marine Advanced Robotics (MAR), adopts a catamaran hull structure that mitigates impact twofold, resulting in up-and-down vibrations caused by waves. The hull is designed to absorb waves, and this USV is used regularly for marine exploration that requires precise hull posture. Google used this to acquire coastal view images in San Francisco Bay, and the Port Authority succeeded in measuring the precise water depth of the Raccoon Strait inside San Francisco Bay by mounting Kongsberg’s M3 MBES sensor [56].

#### 2.1.3. USVs by Country

In addition to the United States, which is leading the way in USV development, many other countries are actively working to advance defense and civil technology. The following discusses USV development by country:

i. United States

In the mid-1990s, the United States developed a high-speed target USV for military training called ‘Owl’ and a multipurpose USV called ‘Roboski’ [45]. In the late 1990s, Corp. Navtec equipped OWL with a jet ski chassis and side scanner equipped with a video camera and improved its stealth function and loading capacity so it could operate in the Persian Gulf. It also developed an advanced modified unmanned ship ‘SPARTAN’, 7–11 m in size with a loading capacity of 1.5–2.5 tons, that was capable of military special operations [2]. After this, the US Navy launched a new unmanned ship program in 2003. In this program, a low-cost unmanned ship specialized in intelligent surveillance and reconnaissance was developed, as was a small weapons attack trainer (SWAT) equipped with 25 mm, 50 k, M-60, and close-range shooting capabilities.

ii. Israel

Israel developed ‘Protector’ in 2003 [46], which is smaller than the US-developed Roboski. ‘Protector’ of the Israel Rafael weapon, ‘Stingray’ of the Israel Elbit systems Ltd., and ‘Katana’ of the Israel aerospace industries (IAI) are typical USV technologies developed by their domestic companies [53]. Rafael, an Israeli state-run defense company, recently completed a sea launch test of a spike ER missile using a Protector in 2017 [57].

iii. Japan

Japan has developed an unmanned high-speed ship and two unmanned ships. The Unmanned Marine Vehicle High-Speed (UMV-H) [58] is a compact 4.44 m long unmanned and manned vehicle that uses water jet propellant but has been developed to be equipped with equipment such as underwater cameras and sonars. Unmanned Marine Vehicle Ocean type (UMV-O) [58] is used by the Japan Science and Technology Agency and is mainly used for monitoring and research of physical parameters of the ocean and atmosphere, biogeography, and chemistry.

iv. The United Kingdom

In the UK, an unmanned ship ‘Springer’ that can operate in shallow water was developed for water quality inspection and environmental investigation [41,59,60]. QinetiQ Ltd. in the UK has developed an unmanned ship called ‘MIMIR’ for using Iraq War in 2003 that maximizes access to shallow water and opportunities for wireless data acquisition by using a remote control system [42]. Additionally, AutoNaut currently produces USVs that are driven by wave movement. Renewable energy allows for weeks of ocean missions without carbon fuel emissions or costs [61]. The USV ‘Jura’ was tested as an illegal, unreported, and unregulated (IUU) surveillance platform in April and May 2016 [61]. A USV called ‘Islay’ was tested for ASW.

v. Norway

One notable project in Norway is the YARA Birkeland [62], an autonomous container ship being developed by the Norwegian fertilizer company YARA in partnership with the technology company Kongsberg. The YARA Birkeland is designed to transport fertilizer from YARA’s production facility in Porsgrunn, Norway, to the nearby port of Larvik. The ship will have a capacity of up to 120 TEUs (twenty-foot equivalent units). Other Norwegian projects include the development of autonomous ferries by the technology company Massterly [63], which are being tested in the Oslofjord region; and the development of autonomous underwater vehicles by the Norwegian University of Science and Technology, which are used for oceanographic research and exploration.

vi. Spain

Sentinel 2.0. was developed by Ibercisa in Spain [63] and is an autonomous surface vessel designed for marine surveying, monitoring, and inspection. Sentinel 2.0 has a modular design, allowing it to be configured with different sensors and payloads depending on the specific mission requirements. The vessel can be used for a variety of applications, including environmental monitoring, offshore oil and gas operations, and marine research. Other Spanish projects include the development of autonomous underwater gliders by the Spanish National Research Council, which are used for oceanographic research and monitoring.

vii. China

The country’s Ministry of Industry and Information Technology has identified autonomous ships as a key area for development, and several Chinese companies are developing unmanned ship technology. Notable Chinese projects include the development of autonomous cargo ships by the shipping company COSCO Shipping Lines [64], which tested on a route between China and Europe; and the development of a fleet of autonomous patrol boats by the technology company Yunzhou Tech [65], which are used for maritime law enforcement and security operations.

viii. Poland

The unmanned surface vehicle USV ‘EDREDON’ was built in Poland [66]. The USV was developed by researchers from the Naval Academy in Gdynia and Gdansk University of Technology as a result of the research project ‘Unmanned swimming platforms for the protection of national sea services’. The vehicle is remotely controlled and equipped with devices and sensors that allow for the flexible implementation of various tasks. Its modular construction allows for a vast scope of applications and changes in the purpose of use. The vehicle can be extended for military applications by installing additional modules, such as a remotely controlled machine gun, depth charge launcher, an unmanned underwater vehicle, a disposable underwater vehicle against floating mines, a search light, and towed sonar.

### 2.2. Unmanned Underwater Vehicles (UUVs)

A UUV is a type of submarine that performs underwater missions. Advantageously, UUVs can be built in a much smaller size and at a lower cost than can traditional submarines because they are unmanned. However, operating UUVs in highly volatile underwater environments is a formidable challenge [67]. Because the UUV performs its mission underwater, it must maintain the scheduled route even in environments that are difficult to operate owing to high currents or heavy water pressure. Its devices are easily corroded because of seawater. Moreover, the utilization of radio signals and satellite navigation systems is limited.

Although it is evident from the history of UUVs that the first UUV was an ROV, it is still unknown whether the first UUV was a programmed under vehicle (PUV) developed by Luppis-Whitehead Automobile in Austria in 1864 or a POODLE developed by Dimitri Rebikoff in 1953 [68]. The early UUV market was dominated by the ROV and was developed based on commercial needs. The University of Washington first developed a UUV in the 1950s called the SPURV, which could dive up to 10,000 feet deep [1]. In the 1960s, SPURV was used to recover lost equipment, and an ROV called CURV was used to recover an atomic bomb off the coast of Spain. In the 1970s, it was used to rescue crew members of submersible deep-sea submarines, but its primarily function was underwater data collection and transmission. In the 1980s, CURV discovered the remains of the Titanic and the World War II battleship Bismarck. In the early 1990s, attempts were made to use UUVs in the military to discover, approach, and neutralize mines without the enemy’s knowledge. In the 2000s, UUVs were first used in combat environments. The United States used REMUS UUVs to clear the area around the port of Umm Qasr during Operation Iraqi Freedom in 2003 [69]. UUVs are utilized in ISRs, MCMs, and ASWs for military purposes, similarly to USVs. For this reason, they have recently been connected and applied to manned/unmanned surface ships. Furthermore, the UUV, which has precise underwater navigation, is actively engaged in research in the private sector, for example, in collecting data from living organisms and safely exploring the deep sea. Currently, developed countries with strong military power continue to develop and operate UUVs with precise underwater navigation capabilities.

#### 2.2.1. UUV Type

In 2004, the US Navy’s UUV Master Plan divided UUVs into four classes based on weight and durability [70,71]. The UUV types and brief information about each type are presented in Table 3, and a supplementary explanation for each class is presented as follows:

#### 2.2.2. Application

Most roles of the UUV, including ISR, MCM, and ASW, are almost identical to those of the USV. However, a UUV can be used to secretly transport payloads (e.g., cameras, sensors, sonar, LiDAR, and others) because it operates even in shallow water. UUVs are effective for gathering information owing to their small size and ability to operate easily in shallow waters. UUVs also play an important role in time-critical strikes (TCS) because they can stealthily approach and attack targets underwater, thereby minimizing the enemy’s reaction time. The list of UUV missions is as follows:

i. ASW

For ASW, UUVs are used for ambushes, enemy patrols, and covert site attacks. Additionally, they can enter enemy positions, gather information, send data to allies, or attack enemy submarines and traps.

ii. ISR

ISR is the basis of ASW, which includes detecting and tracking sounds from enemy submarines during operation. The navy can have a general view of the battlefield and observe whether the enemy is coming by maintaining a surveillance system.

iii. MCM

UUVs are used to detect, avoid, and remove mines buried underwater to keep the fleet safe. The detection sensors mounted on the UUV are used to check whether the mine is present, and the missile or other UUV mounted on the USV or carrier is used to neutralize it to create or avoid a path.

iv. Network and communication

Underwater sensor nodes, which make up the underwater wireless sensor network (UWSN), can stably transmit various types of underwater information, for example, water temperature, salt, and dissolved oxygen on land, by observing the environment in real-time and are utilized in various fields, such as those involving earthquakes and tsunamis [75]. The collected data are transmitted via a maritime communication buoy to satellites or land. A sonobuoy that can transmit data directly from the water to the surface has been recently developed and used as an underwater sensor node [76,77]. Connectivity has become an important issue in underwater sensors and mobile ad hoc networks, as data are passed through collaboration between the nodes in the network. The AUV contributes significantly to network resilience in this case. This is because AUVs can be used to place additional nodes in areas with physical communication gaps [78]. An AUV is positioned with a normal underwater sensor node if it is used as a node in an underwater network. The sensor nodes are deployed all at once or split into multiple deployments to recover the networks reduced by the involuntary movement of underwater sensor nodes for ocean currents. There are several ways to recover a network. The classical method restores wireless ad hoc and sensor networks using the partition detection and recovery algorithm (PADRA) protocol [79]. The methods described in [80,81] restore the network by depth adjustment of the underwater sensor nodes using centralized topology control (CTC) and distributed topology control (DTC) algorithms, and the final method restores the connection using an AUV [78,82]. In [82], the AUV acted as a bridge for locating and delivering important information such as messages or time on disconnected nodes and then identifying important communication gaps between underwater sensor nodes. Military and civilian organization use it for inspection/identification, payload delivery, and oceanography.

v. Underwater structure maintenance

Demand for underwater robots for functions such as construction and maintenance of underwater tunnels, maintenance of offshore piers, and installation and operation management of underwater oil field facilities is increasing significantly. In the early 2000s, the University of Hawaii’s semiautonomous underwater vehicle for intervention mission (SAUVIM) project in the US and the ALIVE project in Europe developed automation technology for underwater work including object recovery using manipulation attached to underwater robots. In Europe, the concept of intervention AUV, which automates tasks requiring human control, has been proposed, and core research has been conducted through the Trident and Triton projects. The University of Girona 500 AUV is a compact three-cylinder AUV developed as part of the Triton project. Subsequently, research for the usefulness and verification of technology by the Persistent Autonomy Through Learning Adaptation Observation and Re-Planning (PANDORA) project and the Twinbot project, in which two robots complete missions through cooperative actions, was recently conducted [83,84].

#### 2.2.3. UUVs by Country

Currently, many countries are promoting projects to prepare for future unmanned underwater warfare, and several private companies are actively pursuing UUV development for underwater research. Representative country UUV development and the UUV these countries have developed are introduced below. Moreover, Table 4 shows further more information on the developments in each country.

i. United States

UUVs are playing an increasingly large role in US naval operations and are expected to enhance the underwater power advantage of the US Navy. Bluefin Robotics has continually developed the Bluefin series [85]. The Boeing Company invented a long-term mine reconnaissance system (LMRS) AUV for the military and Echo Ranger AUV for civilians [86]. Many companies continue to actively develop UUVs in the United States.

ii. The United Kingdom

The UK started the Autosub development project in 1988, and an underwater robot for ocean exploration, Autosub-I, was developed in 1996, after which the 6000 m class Autosub 6000 was developed in 2007 [87]. As a military underwater robot, Talisman was developed by BAE Systems and demonstrated in 2005 [88].

iii. France

In France, ECA developed the first acoustically controlled 6000 m AUV Epaulard in 1981; developed the Alister-MDV, an AUV for demonstration of mine search and covert reconnaissance technology in 2002; and developed a new minesweeper, the K-Ster [89]. In 2008, it announced the heavy torpedo type UUV with the concept of submarine launch and recovery.

iv. Germany

Germany developed SeaFox [90], an expendable minesweeper for mine neutralization, for the first time in the world, and developed and operated various unmanned submersibles such as Maridan UUV-based mine countermeasure SeaOtter, SeaWolf, and UUV-DeepC independently or in the form of a consortium with countries in the EC [91].

v. Japan

Japan developed the Dolphin 3K [92] and used it to conduct deep-sea observations of hydrothermal deposits, photosynthesis-independent biological colonies, and new tectonic plates. In 1996, Japan developed the R1 underwater robot equipped with a closed-circuit diesel engine, and in 1998, it developed the long-range unmanned submarine Urashima powered by a fuel cell. JAMSTEC has been developing the MR-X1 AUV for 4200 m deep sea exploration since 2000 [93].

vi. Sweden

Sweden has a strong presence in the UUV industry with several companies and institutions developing and producing UUVs for various applications. Saab Seaeye produces a range of UUVs, including ROVs and AUVs, with examples being the Falcon ROV, Sabertooth AUV, and the Seaeye Tiger ROV [94] [95]. Their vehicles are used in various applications, such as oil and gas exploration, oceanographic research, and underwater inspections. The Swedish Defense Research Agency (FOI) is a government agency that conducts research and development for national defense and security. Collaborating with companies, they have developed several UUVs, including AUV 62 (AUV62-MR/AT), which is designed for submarine hunting or applications such as mine countermeasures and environmental monitoring [94].

vii. Norway

Kongsberg Maritime, a Norwegian company, also has a significant presence in Sweden and produces UUVs, including HUGIN and Munin which are remotely operated towed vehicles (ROTVs) [96,97]. Their vehicles are used in applications such as seabed mapping, pipeline inspection, and environmental monitoring.

viii. Korea

Korea developed SAUV for research that can be used as a military MDV in collaboration with Ocean Electric Industries Co., Ltd in Chattogram, Bangladesh in the 2000s, and the Korea Ocean Research and Development Institute (KORDI) developed an autonomous navigation unmanned mine processor (MDV) as an ACTD task. In 2008, Daewoo Shipbuilding & Marine Engineering Co., Ltd. in Geoje city, Korea and Daewon Mechatronics., Ltd. in Changwon city, Korea developed a cleaning robot that cleans the hull of the ship underwater. In 2009, KORDI developed the UUV ‘ISiMI’ for shallow sea operations, and similarly, Samsung Thales developed the BOTO, an underwater robot for marine exploration, in 2011.

**Table 4:** Countries, companies, and their work for developing UUVs [98].

Country	Company	Work
USA	Bluefin Robotics	Developing and supplying main AUV (Odyssey AUV, Bluefin 9, 12, 21 dia AUV)
	Boeing	Developing LMRS AUV (military), Echo Ranger AUV (civilian)
	Hydroid	REMUS AUV technology development (WHOI Source development, License)
	iRobot	Ranger UUV (Sea Glider)
	Lockheed Martin	RMMV, BAE Archerfish EMDV production and submarine launching development
	Oceaneering	ROV production/operation and development of Echo Ranger Vehicle AUV
	Ocean Server Technology	Developing a small, light, low-cost AUV
	Teledyne Webb Research	Designing and making the Apex profiling float, Slocum glider, and Discuss glider
UK	ASV Ltd.	Designing a surface, semisubmersible/underwater towing platform
	BAE Systems	Developing an AUV (multirole platform) and airborne EMDV Archerfish
	Go Science	Developing the Autotracker system
	Hydro-lek	Producing a manipulator for ROVs
	Saab Seaeye	Designing a ring-wing AUV
France	ACSA	Developing underwater navigation/GPS receiver/acoustic localization system and gliding AUV
	Cybernetix	Developing ALIVE AUV and SWIMMIER
	DCNS	Developing the ASM-X AUV
	ECA	Producing the RAP ROV (MCM) and developing a new concept MCM AUV for the navy
	Thales Underwater system	Producing a towed MCM sonar/PVDM and developing an MCM AUV with ECA
Germany	Atlas Electronik	Developing marine electronic system supply
	Alstrom Schilling Robotics	Developing the ROV Quest 4000
	Herion Systemtechik Gmbh	Developing David
Canada	International Submarine Engineering (ISE)	Developing the complex swimmer AUV/ROV
	Marport Deep Sea Technology	Developing a sound monitoring system for deep sea fish
Japan	Mitsui Engineering & Shipbuilding (MES)	Developing the Aqua-Explorer AUV
Australia	Woodside Energy	Developing AUV for Exploration and production of undersea gas lines and inspection pipeline

## 3. Core Elements of UMVs

The core technologies required in UMVs are discussed in this section. Many companies are working diligently to add more features to these technologies and improve stealth capabilities.

### 3.1. Sensors

Small and high-performance light sensors have been continuously developed since the early 2000s. In USV and UUV operations, depth measurement equipment plays a crucial role in enabling the vehicles to navigate and perform tasks accurately. Through product generalization, both USVs and UUVs have detachable sensors in the form of modules used whenever necessary, enabling them to be used in many ways. Presently, onboard sonar, photoelectric, and other sensors are mainly used to search for the range of a specified water area to realize the detection, identification, location, and demining of mines. Sonars are commonly used to measure depth in water by emitting sound waves and measuring the time taken for the echoes to return. Meanwhile, light sensors are used to detect and identify underwater objects and can also be used to estimate depth based on the amount of light that penetrates the water. These sensors are often detachable and can be used in different modules as required. The key details regarding the sensors used in USVs and UUVs are included below.

#### 3.1.1. Sonar

Sonar is a device that determines the direction and distance of a target underwater using sound waves. Sonar is advantageously used in underwater imaging conditions [99], and are more suitable compared with optical sensors that are limited by imaging distance [100]. Therefore, acoustic sonar information is essential in the case of USVs and UUVs [101].

In addition to its advantages in underwater imaging, sonar technology can also provide valuable information for navigation and safety purposes in unmanned marine vehicles. Sonar systems can be used to detect underwater obstacles and potential hazards, such as mines or underwater structures, which can help to prevent accidents and damage to the vehicle. The two main types of sonar used in unmanned submersibles are front-looking sonar (FLS) and side-scan sonar (SSS). FLS is used to detect objects in front of the vehicle, while SSS can be used to create detailed images of the seafloor and surrounding area.

One challenge with sonar technology is that it can be affected by environmental factors such as water temperature, salinity, and pressure, which can affect the accuracy and reliability of the readings. Additionally, sonar systems can be vulnerable to interference from other underwater sources of sound, such as marine life or other vehicles. To address these challenges, researchers are working to develop advanced sonar systems that are more robust and can provide more accurate and reliable information in a range of environmental conditions.

##### Side Scan Sonar and Synthetic Aperture Sonar

**Figure 3:**
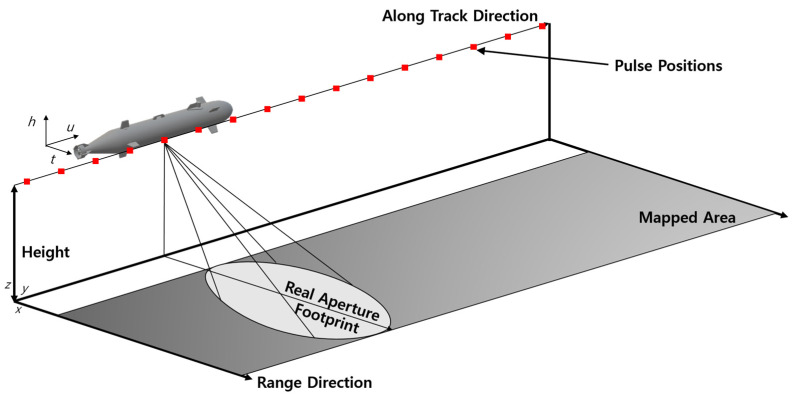
Illustration of the side scan sonar systems [102].

**Figure 4:**
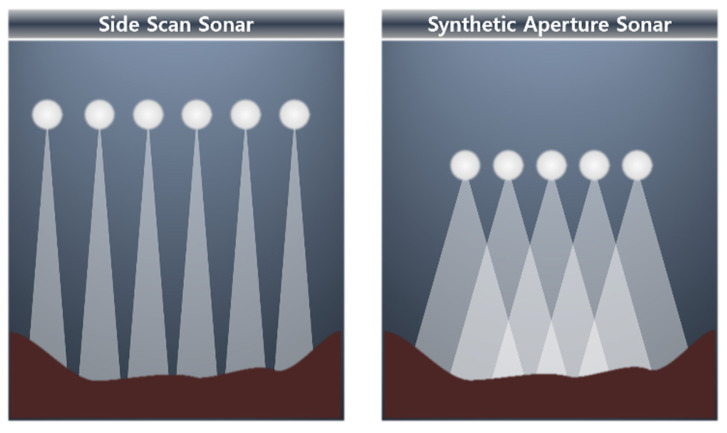
Side scan sonar (**left**) and synthetic aperture sonar (**right**) [106].

The side scan sonar (SSS) is a device that displays the seabed as a two-dimensional image using sound waves, as shown in Figure 3. SSS can effectively create images of the seabed and is mainly used for surveying the seabed topography. The image generated by the SSS is different from that generated by an optical image, which is a general photograph. SSS images are represented by highlights made by the part where sound waves hit an object and are directly reflected and by shadows created by the part where sound waves do not reach the object [102]. The resolution of the SSS is low owing to the various noises present in water [101], and its typical operating range is around 100 to 200 m. Thus, synthetic aperture sonar (SAS) has been developed to increase the resolution of scanned images in recent years. SAS is a technology that applies the idea of synthetic aperture radar (SAR) to sonar [103]. SAS increases the resolution by combining multiple openings through signal processing, and this overcomes the physical limitation of the array length of SSS by more than 10 times compared with that of SSS [104]. Specifically, given the maneuvering information of the sonar, the resolution can be increased as if it were a sonar image obtained using a physically large aperture by adding past measurements to the present measurement to create a sonar image. Primarily, SAS is mounted on UUVs for exploration, and the underwater communication of the exploring regions is normally limited. Therefore, high-resolution images cannot be sent to operators. Therefore, SAS images may not be visible until exploration is complete [105]. A schematic description of the difference between SSS and SAS is shown in Figure 4.

##### Front-Looking Sonar

A front-looking sonar (FLS) is a type of sonar used in marine navigation systems that is designed to detect objects in front of a ship or UMV (unmanned marine vehicle) [107]. An FLS device typically weighs between 30 and 80 pounds and has a power consumption of around 15–30 watts. FLS can detect objects at distances of up to several hundred meters, depending on the size and shape of the object and the acoustic properties of the surrounding water. Some FLS systems can also be integrated with other navigation systems, such as radar or GPS, to provide more comprehensive situational awareness and obstacle-avoidance capabilities. As sensor technology continues to advance, it is expected that FLS systems will become even more accurate and energy efficient, enabling them to be used on an even wider range of vessels and UMVs. FLS works by transmitting an acoustic signal and then measuring the time it takes for the signal to bounce back from an object in front of the vessel. This enables the system to detect and locate obstacles or other objects in the vessel’s path, which can help to prevent collisions or other accidents.

#### 3.1.2. Doppler Velocity Log (DVL)

Since radio waves do not pass underwater, the location cannot be observed using a global positioning system (GPS). Therefore, navigation sensors using ultrasonic observation systems are mounted on various marine vehicles and used for position estimation. Among them, Doppler velocity log (DVL) [108,109], a representative auxiliary navigation sensor, is a representative speed measurement system that uses the Doppler effect. It calculates the relative speed between the water area and the device using the Doppler frequency change of the sound signal which is reflected and returns from the seabed. 

#### 3.1.3. Gyroscope

A gyroscope (gyro) is a piece of equipment that utilizes the earth’s gravity to determine its direction [110,111]. The gyro sensor detects an angular speed per unit of time by measuring a rotational speed around a specific axis and is generally expressed in deg/s (per second). There are three types of gyros: rotational mass, vibration, and optical. The rotational mass gyro uses a spinning wheel to resist any attempt to change the direction of its axis of rotation, while the vibration gyro measures angular velocity using a vibrating element. The optical gyro, on the other hand, utilizes the interference of light waves to measure rotation.

Gyroscopes are commonly used in navigation systems, such as airplanes, spacecraft, and ships, to help determine orientation and maintain stability [112]. They are also used in various other fields, including robotics, virtual reality, and smartphones, where they detect and measure the angular velocity and orientation of the device [113]. One significant advantage of gyroscopes is their high level of accuracy, as external forces or disturbances such as vibration or changes in temperature do not affect them. Additionally, they are compact and lightweight, making them suitable for use in small devices.

However, gyroscopes are sensitive to mechanical wear and tear, which can affect their accuracy over time, and can drift, requiring frequent calibration. Despite these limitations, gyroscopes play a crucial role in various technological applications, enabling accurate measurements of angular velocity and orientation and facilitating precise navigation and control.

#### 3.1.4. Inertial Navigation System (INS)

The rotational angular velocity and linear acceleration of the vehicle are measured by a gyroscope and accelerometer called an inertial sensor, and with these outputs, information on the vehicle’s current position, speed, and attitude relative to the reference navigation coordinate system is provided without external help [114]. Therefore, the INS can avoid signal disturbance or signal detection from the outside and is not subject to weather or time restrictions at all. The INS consists of a gimbaled INS and strapdown INS: (1) The gimbaled INS is mounted on a stabilized platform that physically isolates the inertial sensor from external rotational motion. A virtual analytical system is mathematically defined by the navigation computer by firmly mounting the inertial sensor directly on the fuselage [115], and (2) a strapdown INS calculates navigation information on the platform [116].

**Figure 5:**
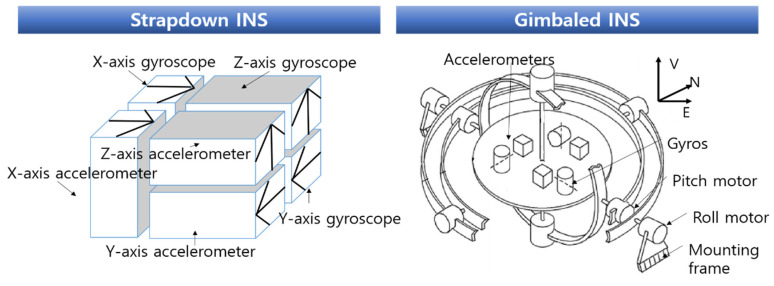
Strapdown INS Schematic (**Left**), Gimbaled INS Schematic (**Right**).

In addition to this, various sensors for research and defense projects such as pressure sensors and temperature sensors can be mounted. Figure 5 shows the schematic of the strapdown and gimbaled INS.

#### 3.1.5. Magnetic Sensor

Magnetic sensors are frequently mounted for security and military applications, such as detection, identification, navigation, positioning, and antitheft systems of ferromagnetic and conductive objects, and are used to detect metals such as mines and torpedoes [117,118].

### 3.2. Battery

An energy source capable of driving a propulsion engine is required to move a key surveillance area over a long period of time underwater or on the surface, or to maintain a position under the influence of rapid currents. Various energy sources, such as zinc-silver batteries, lead acid batteries, Li-ion batteries, and lithium polymer batteries, are being used in unmanned submersibles. However, additional research and development on the energy source are essential for the long-term operation of UMVs because the operation time of the unmanned submersible is significantly affected by the power consumption of the energy source.

Several previous studies have attempted to overcome this problem. Liquid Robotics has developed a wave glider (USV) that utilizes the energy of waves [119]. Additional solar panels have been used to power onboard electronic devices to solve battery problems. Innovation Inc. developed aluminum seawater batteries for micro-UUVs in exclusive partnership with Open Water Power Inc. in Boston, US [72]. They claimed that the durability of micro-AUVs can be extended to more than 10 times their current production capacity using this new battery technology.

If the battery issue is resolved, it could transform ocean science and defense by replacing expensive stationary mooring, facilitating autonomous movement, and allowing for the reconnection of disconnected devices.

## 4. Intelligence

Automation and autonomy are important in unmanned systems. In the case of an manned system, the operator directly runs the vehicle and senses the surrounding environment; therefore, it is possible to respond immediately. However, all changes in the environment are detected only by the onboard sensing device when an unmanned system is used, and situation identification and response are relatively slow because the environmental status is transmitted through wireless communication. Therefore, unmanned vehicles have begun to strive for automation [120]. This involves the development of long-term targeting and control navigation for mission performance, with the aim of maximizing operational efficiency by communicating with and complementing various types of equipment.

Recently, research has expanded beyond automated driving, in which the machine automatically controls and operates as it is set by a human, to vehicles equipped with autonomous driving that operate independently without the help of humans or others, owing to the increase in computing power and miniaturization of electronic parts.

However, unmanned vehicles are in the transition period from automated to autonomous systems. Next, we discuss the recent status of automated and autonomous systems for UMVs.

### 4.1. Automated Systems

Currently, unmanned vehicles require constant operator supervision to avoid obstacles. The operator’s physical strength and manpower are continuously required because most unmanned vehicles are still directly controlled by a coordinator through a wireless connection from a distance. The International Maritime Organization (IMO) categorizes four stages of autonomy for maritime autonomous surface ships (MASS) to define the autonomy of the USV, as shown in Table 5.

This step can be applied to demonstrate that unmanned systems are at least as safe as manned vessel systems and to provide appropriate situational awareness to shore control centers (SCCs). However, the development of a fully autonomous USV continues because human intervention is unavoidable when USVs are in emergencies [122,123]. The US Navy has developed the Anaconda 2.0 USV, which can be operated remotely in an unobstructed area; however, an autonomous USV equipped with full artificial intelligence (AI) is still under development [53].

### 4.2. Autonomous Systems

In practice, unmanned vehicles with complete autonomy are unavailable. Therefore, technology is needed to minimize the loading time of the pilot and the response time of the USV and maintain reliable performance to semiautomatically control and send data to the operator for direct control even if an autonomous system is installed in the USV [124]. In the case of the UUV, unlike in ground or air environments, navigation information or sensors using location recognition are used in environments that are difficult to recognize; therefore, engineers exerting considerable effort to integrate intelligence to perform underwater unmanned autonomous driving without using communication.

The UUV should be able to cope appropriately in various marine environments while operating from the starting point to the destination along a given route, especially if the remote control is not operating beyond sight. To complete a specific task, the ship or obstacle encountered during navigation should be automatically avoided without the need for the manipulation of the control unit on the land; the object should be detected, analyzed, and reacted to on its own; and the ability to recover itself when a failure or a system error occurs. Furthermore, unmanned vehicles should have core technologies, such as route planning, path control, location estimation, and learning reasoning, for autonomous operation [125].

In addition to the above-core technology, it is also important to recover the UUV and the USV. Both the USV and the UUV are sensitive to slight changes in sea conditions when launching automatically. Therefore, it is necessary to study the information on the sea state through sensors, determine the route, and dock safely. Furthermore, many technologies, such as those of detection, communication, hydrodynamics, maneuvering, robotics, control, data fusion, and high-level fault-tolerant docking control strategies, must be integrated to enable accurate docking contact strategies [126,127]. Recently, swarm robotics with swarm intelligence has been actively studied. This is discussed in detail in the following section. In this section, we discuss only the control, detection/classification/discrimination/characterization, and ISR, where AI is applied to autonomously move the USV and UUV. Table 6 and Table 7 show UUVs and USVs single intelligence algorithms.

#### Control USV

**Figure 6:**
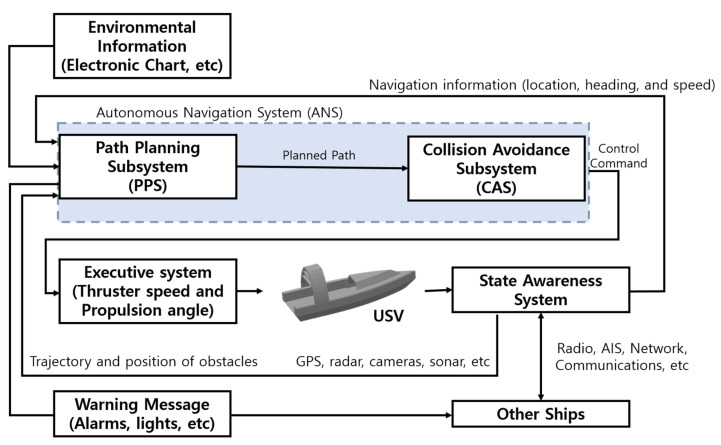
USV autonomous navigation structure [127].

Figure 6 shows an example of a USV autonomous navigation algorithm comprising sensors and systems. Advances in deep learning and accumulated experience have led to the development of sensors and systems for USV direction, exploration, control, telemetry, propulsion, and path planning. Many institutions are steadily participating in USV development projects based on this, and the representative projects are summarized, as shown in Table 8.

In addition to large projects, sonar, photoelectric, and other sensors are used in many places to search for the extent of designated water bodies to enable identification, localization, and demining. It is possible to search for coverage using a circuit algorithm [140,141] for target waters with regular shapes and known environments, and frontier-based methods [142] and improved algorithms are mainly used [143,144,145,146] for complex unknown environments with dynamic obstacles. Methods for measuring the depth of the water against the natural sea using autonomous/unmanned irradiation lines operating on global navigation satellite system (GNSS) measurements or satellite images have also been developed [147].

i. Controlling UUVs

**Figure 7:**
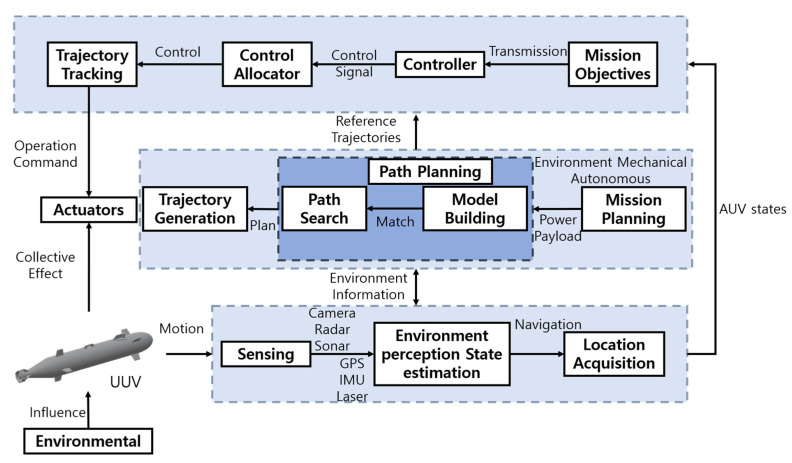
UUV autonomous navigation structure [147].

AI technology is required to manipulate the UUV body by adjusting the speed for surge, sway, heave, and the angular speed for pitch and yaw when controlling a UUV. UUV control is a subject that is being intensely addressed [147,148,149,150,151,152,153,154,155,156,157,158,159,160,161,162,163]. Adjusting the UUV body underwater is required, where communication is difficult, and the UUV should be recovered well at a limited communication bandwidth. The algorithm for navigating an autonomous UUV is illustrated in Figure 7.

A method has been developed to actively achieve self-rescue control by adjusting autonomous AUV motion control for each fin using a deep deterministic policy gradient (DDPG) to improve the performance of autonomous navigation of UUVs [149]. Algorithms are also being developed to avoid underwater obstacles for the autonomy of AUVs [150]. Studies to design algorithms to efficiently move and navigate underwater with a route using a UUV have been conducted [148]. Several algorithms are being developed to patrol underwater boundaries using UUVs [151,152]. However, it is difficult to monitor underwater boundaries because the UUV uses only scalar sensors and only acoustic and asynchronous communication (surface RF). Thus, the Monterey Bay Aquarium Research Institute has developed a snake algorithm and UUV gas methods for underwater boundary monitoring [153].

ii. ISR

Unmanned vehicles primarily perform border patrols, surveillance, and strikes in major areas when they perform surveillance and reconnaissance missions. A border area is observed along the border and periodically examined using single or multiple unmanned vehicles when patrolling it. The task is to observe and attack the moving object while avoiding torpedoes in the event of an exhibition or emergency. This mission is basically a problem that requires minimizing the distance while efficiently detecting or avoiding obstacles; thus, a complex algorithm with intelligence is required [154,155,156,157,158]. Recently, methods of setting the shortest path by combining neural networks and maintaining a swarm when using multiple UMVs have attracted attention. In [159], Hui et al. proposed an adaptive navigation algorithm that applies in-depth learning so that the AUV can accurately search, considering the measurement deviation of microelectromechanical system (MEMS) sensors. It uses deep learning to generate low-frequency localization information to correct search errors and uses the χ^2^ rule to avoid interference from Doppler velocity log (DVL) outliers when the DVL measurement fails. Zhu et al. proposed a current effect-eliminated bio-inspired neural network path planning (CBNNP) algorithm in [157]. It is a neural network-based algorithm that combines distance and direction optimization with neural network theory to infer the shortest path and avoid all possible collisions. It also acts as an algorithm, and adjusting components based on parallelogram law which offsets the deviation caused by current influence has been proposed. Additionally, an algorithm for avoiding collisions based on the speed of several USVs was proposed in [157].

iii. Detection, classification, discrimination, and characterization

Autonomous detection technology is a key technology for UUVs and USVs, which can use sonar to collect data and detect targets on their own. The intelligence being developed to automatically detect and track targets has not yet followed the experience of skilled technicians. Zhou et al. proposed an order truncate-average-constant false alarm rate (OTA-CFAR) algorithm that fuses the OTA algorithm in [160], which is used to equalize background noise and eliminate captured averages. They also proposed CFAR, which is a signal processing method that provides thresholds for detection methods in an auto-sensing system and minimizes the impact of background noise and interference on the probability of false alarms in the system. Detection techniques using feature extraction, matched field processing (MFP), and intelligence were proposed in [161]. Intelligent target detection techniques include methods to increase the SNR and detection coverage by combining machine learning and conventional signal processing and autonomous cognitive detection techniques focused on adaptive recognition and computation based on intelligent cognitive processes [162,163].

### 4.3. Others

A towed underwater platform (TUP) connected to a USV is being developed to compensate for navigation errors in the underwater environment and overcome communication limitations caused by battery problems and long-term operation [164].

In addition to adding a sensor, it is also possible to use both mechanical and material approaches. There is a way to use an anechoic tile or reduce radiated noise to maintain the stealth function of the UUV [165]. Especially in the case of bionic AUVs, it is difficult to determine whether they are enemies when they try to reduce vibration noise or utilize sound-absorbing materials because they have only mechanical and hydrodynamic noise.

## 5. Swarm and Cooperation between Unmanned Vehicles

Despite various studies being conducted in each field, there are still many challenges to be resolved in order to autonomously perform missions in the changing marine environment. It is still more difficult to communicate in the underwater environment than it is on Mars, which is 70 million km away, and as the use of drones expands, the types and complexity of missions performed will increase. Connecting tasks that are difficult or impossible to do with a single device and performing them simultaneously through collaboration can drastically save time and money.

These homogenous coordinated operations are based on the development of communications and networking technologies, navigation and acoustic exploration technologies, and multidrone mission planning technologies. This concept has expanded from a simple cooperative mission where the exploration area is divided into balls, to a complex mission where multiple vehicles with different functions cooperate. Through mutual cooperation of n underwater drones, work that is difficult to perform with individual drones has more than n times the irradiation efficiency.

To achieve this goal, cooperative algorithms are being actively devised, and vehicles can be properly implemented using cooperative control and cluster control [4,5]. In addition to this, many efforts are being made toward using cooperation in various places. Table 9 and Table 10 show the works related to multi robot and swarm intelligence:

### 5.1. UUV–UUV Cooperation

**Figure 8:**
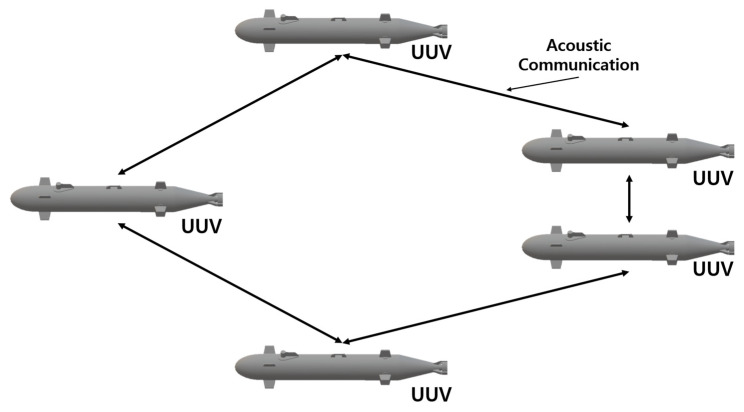
UUV swarm network [173].

There have been studies on cooperation between multiple homogeneous UUVs to solve task assignment problems and avoid underwater obstacles to operate in unknown environments. Wu et al. [173] attempted to solve the task assignment problem using three types of UUVs: portable, lightweight, and heavyweight; they perform three different work types: detection, tracking, and structure. By improving consensus-based bundle algorithm (CBBA)-based algorithms, the proposed that extended CBBA (ECBBA) and dynamic ECBBA (DECBBA) can solve the task assignment problem via operation in two iterative steps: task bundle construction and conflict resolution by improving consensus-based bundle algorithm (CBBA)-based algorithms. The first is ECBBA, which continues to update the assignment process for the static task allocation algorithm, and each UUV continues to add tasks to the task bundle until no more tasks are added or the upper limit of the number of tasks that can be completed is reached. The second is DECBBA, a dynamic task allocation algorithm that changes the entire allocation process from individual time steps to multiple static task allocations to convert the previous static task allocation problem into a dynamic problem. Figure 8 presents a schematic of the UUV swarm network used in [173]. In addition, Yan et al. [174] tracked multiple AUV formation controls and AUV formation trajectories using the proposed underwater obstacle avoidance algorithm when obstacles were detected using multiple AUVs. Simulations were performed, and several AUVs succeeded in tracking obstacles in a three-dimensional space assuming that reliable obstacle information could be obtained through sonar or sensor devices. The system uses an improved artificial potential field method (APF) to avoid the obstacle and then returns when an obstacle is detected in the trajectory.

### 5.2. USV–USV Cooperation

The USV is a system that can be effectively operated in an operational environment in which the army, navy, and air force operate simultaneously, so the value of its use in the defense sector continues to increase. The USVs developed thus far are mainly operated alone, so it is difficult to respond immediately and efficiently to battlefield situations. USVs attempt to perform tasks in clusters to overcome these limitations. Song et al. [175] proposed an observation, orientation, decision, and action (OODA) ring structure for defense on the battlefield. The Department of Defense Architecture Framework (DoDAF) operational perspective and system modeling language (SysML) were used to construct the architecture for this mission system to operate the ring. X. Fang et al. [176] proposed a USV multigoal path planning algorithm using S-57 electronic chart data and the adaptive and mutual learning particle swarm optimization (AMPSO) algorithm. The S-57 electronic chart data were analyzed with the ISO8211 rib, a software tool created by the C++ open-source library, and the environment was modeled in a grid form. AMPSO is an algorithm that designs an adaptive adjustment strategy for the inertia weight factor, learning factor, and mutual learning mechanism and improves the PSO algorithm.

### 5.3. UUV–USV Cooperation

**Figure 9:**
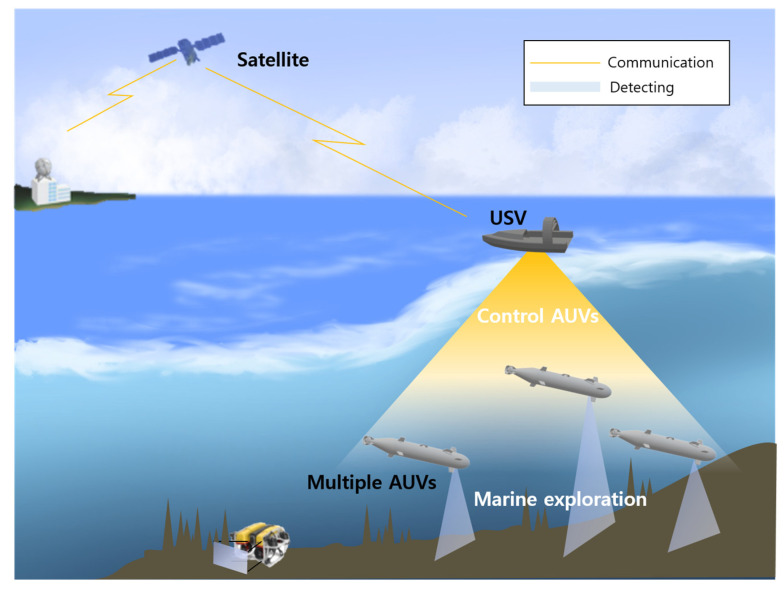
Example of a UUV–USV multiagent system.

When the USV and UUV work together, they can generally be classified into two modes: a single AUV for a single USV and multiple AUV for a single USV. The USV monitors the operation of the AUV; transmits the data it collects to the carriers, research vessels, and headquarters; and receives control commands to operate the AUV. Figure 9 shows an example of a UUV–USV cooperating system. Furthermore, efforts have been made to improve the performance of UUV–USV cooperation [18,19]. McMahon et al. [20] allowed the AUV to achieve its goal by increasing the sum of the rewards so that it can inspect as many targets as possible within the communication range of the USV. A multigoal discrete search was proposed to increase the sum of the rewards. This follows the path determined by the roadmap using motion tree expansion. Kim et al. [19] described a project called Zipangu of the Sea. The USV oversees several AUVs, and the AUV strives to construct a completely autonomous underwater survey device. Four AUVs were used, and a new type of AUV that combined cruising and hovering was utilized.

### 5.4. UAV–UMV Cooperation

**Figure 10:**
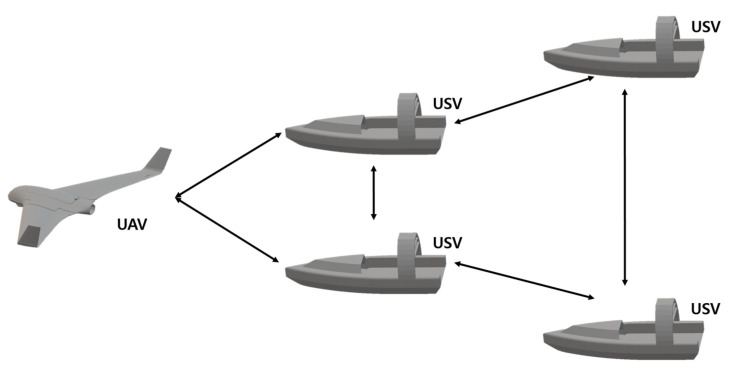
Example of a UAV–USV multiagent system [176].

UMVs can work with UAVs [177,178,179]. Images are mainly collected with UAV cameras so that the UMV can perform tasks smoothly underwater and on the surface. Xue et al. [177] used one UAV and four USVs, the leader–follower consensus method, as demonstrated in Figure 10, and the APF method. The leader–follower architecture describes the relative position between the follower and leader, and converts the large control problem into orbital tracking problems. However, in this experiment, the concept of maintaining a horizontal structure by making the UAV a USV-shaped virtual leader in the case of leader-follower was used because the entire large size is destroyed if the leader fails. Thus, APF methods were proposed together, and these are widely used to deal with the relative distances between agents and help perform large-scale collision avoidance. The ship trajectory controller designed in this study was designed to be applied to unmanned systems with different mechanical characteristics to ensure general universality. The APF function was established to quickly and stably assemble USVs by applying the sliding mode control method and assembling USVs without collision. Furthermore, Shirakura et al. [178] conducted a study on the location and collection of floating marine waste using UAVs and UUVs. Coordinate transformation techniques and a graphical user interface (GUI) were designed to estimate the three-dimensional location of fragments floating on the surface using images from UAVs, and controllers were developed to generate trajectories using teaching playback with feedback to simplify UUV remote manipulation control. In [179], Ross et al. proposed using UUVs, USVs, and UAVs to obtain information about floating targets, for example, unresponsive vessels and icebergs. The value of the water geometry was obtained, and the UUV was examined using sonar to capture the shape and features of the underwater hull using the photogrammetry method with the UAV optical camera. The USV used a central intelligent node to coordinate and control in the middle. Table 11 shows further information on heterogeneous cooperation works:

## 6. Discussion

We have examined the core elements of UMVs and the intelligence applied to UMVs, and many studies have been conducted on cooperation between homogeneous and heterogeneous unmanned vehicles to overcome the limitation of operating a single unmanned vehicle.

A list of supplementary matters in each aspect of UMVs must be considered for their further advancement.

1. It is important to standardize the interface technology and modularization of equipment with advanced mounting equipment to improve the operability of single unmanned vehicles.
2. Underwater vehicles are inevitably limited in communication distance and communication speed due to the physical limitations of underwater acoustic channels. To overcome this, a complex communication system using USVs or buoys combined with RF communication or satellite communication is needed [164,165].
3. An underwater network can be reconstructed by adding sonobuoys in communication using an AUV. In this case, it is necessary to consider the communication and sensing ranges.
4. Submarines have a difficult time recovering UUVs after a mission; therefore, multiple methods for recovering UUVs must also be considered. Many planning methods have been proposed; however, each scheme has its drawbacks [126,127].
5. The use of UMVs in the military is necessary for a wide range of activities; therefore, UMVs must operate through established communication procedures and incorporate the same functionality at the design stage to ensure the availability of effective operating systems in hostile environments.
6. UAVs require the flight skills of naval helicopter pilots as well as reliable USV operation for them to autonomously take off from USVs. Autonomous navigation is necessary to analyze flight conditions and parameters by creating a stable flight pattern based on human experience because it requires sophisticated flight operations.
7. Search strategy, fitness function computations, and memory usage are individual characteristics of an unmanned vehicle that have a significant impact on performance. These individual characteristics can lead to different suitable products for use in problem-solving, exploration, and utilization.
8. The search capability is relatively weak in the case of cooperation between a USV and AUV, and communication is inefficient in the case of a combination of a UAV and AUV. Combined systems that comprise UAVs, USVs, and AUVs can be selected to solve these problems and further increase the efficiency of mission completion.
9. A specific training course should be introduced in this field for design, development, integration, testing, and proper use because unmanned vehicles can affect the economic growth and technological development of each country.
10. The need for and application of the MUM-T system expands according to changes in the social environment, such as a decrease in military service resources and the spread of the idea of valuing human life, and the development of advanced science and technology such as AI and unmanned autonomy. It is expected that by applying AI technology to the MUM-T system, it will be possible to minimize the use of manned forces in the future battlefield and maximize combat effectiveness.
11. Through artificial intelligence enhancement technology, in order to evolve to cooperate with more diverse types of robots suitable for complex missions, new environment recognition, judgment, and control technology and communication technology can be developed.
12. Cooperative operations between unmanned systems such as USVs, UUVs, and UAVs can be subject to issues when communication and sensing are lost. Loss of communication or sensing may cause vehicles to return to a predetermined location or surface as programmed by operators, but if they cannot navigate to a safe location, they may continue to operate in an uncontrolled manner until battery depletion or running aground [165]. Unmanned systems are equipped with redundancy measures such as multiple communication channels, sensors, and control systems to mitigate the risk of communication or sensing loss. Operators may also use preprogrammed responses or contingency plans to minimize the impact of communication or sensing loss.
13. Recently, we have attempted to expand the scope of communication for cooperation with other species. Researchers are also trying to use even 6G in many countries to achieve hyperconnectivity, low connectivity, etc.
14. Many autonomous algorithms are being developed, but there are many difficulties involved with actual application in the field. Thus far, manpower, rather than simple drone deployment, is preferred for high efficiency. There is also a need to develop UVs to protect the human resources deployed.
15. Recently, many drones have been developed for research and civilian use. It may be more prudent to use the knowledge of many countries rather than proceed with a limited project in a single country.
16. For cooperative control of other unmanned vehicles, a clear system must be established. Heterogeneous cooperation requires not only USVs and UUVs, but also a detailed hierarchical control system between heterogeneous vehicles with different functions and platforms [9,184].

## 7. Conclusions

In this study, we explore the evolution of UUVs and USVs, and their employment on the battlefield during recent wars, with a particular focus on UMVs, which have been used in the oceans, along with various other types of unmanned vehicles. In addition to military applications, UUVs and USVs also have numerous civilian applications. UUVs can be used for underwater inspection and maintenance of infrastructure, such as oil rigs and pipelines, as well as for marine biology research and environmental monitoring.

From a hardware perspective, studies are being actively conducted to enhance the battery and underwater communication in UMVs; however, further technological developments are required in these areas. In the mine-countering field, it is expected that the mine removal time will be shortened, and the efficiency will be improved through clustering using SAS technology and multiple unmanned vehicles. Furthermore, in addition to the large UMVs and bio-UUVs that imitate the shape of living organisms, micro-UUVs have been actively developed, which can be easily modified and used for multiple purposes.

Further enhancement of the control intelligence for UMV is required in terms of software. This is because fully autonomous unmanned vehicles have not yet been developed; therefore, they cannot completely replace humans at present. It is important to develop autonomy in a single vehicle, and recently the limitations of distance and operational simplicity have been overcome by the system of collaboration and cooperation; in addition to homogeneous platforms, heterogeneous platforms cooperate to solve various problems.

The past operation of marine systems that depended on large ships is rapidly changing to small unmanned cooperative systems. In order to independently operate a UMV equipped with intelligence, further improvement of the intelligence level of the UMV is needed, but it is also necessary to devise a cooperative method to use multi robot and swarm systems. In addition, no matter how good the sensors and artificial intelligence models are, they cannot function properly if communication between devices is unstable. Therefore, many developments are required for formative control and cooperation in situations where communication is weak.

## Figures and Tables

**Table 1:** Classification and mission of the USV craft type [47].

Type	Description
Semisubmersible (SS) type	A semisubmarine with an average length of 7 m and a speed of 25 kn Less affected by maritime conditions than traditional types of ships Stable and widely used for transportation to supply equipment in the operating area Hard to detect in its SS form, which facilitates armed operations A more complicated design process than that of other vessel types
Conventional planning hull type	General ship type with various hull shapes V-type, modified V-type, and M-type High operational efficiency at a speed of up to 20 kn Large effective load Not complicated in design Low production costs Poor transportation stability Rolling and slam phenomena easily occur under the influence of sea conditions
Hydrofoil craft type	Strongest ability to adapt to sea conditions and good stability Speed up to 40 kn Outstanding operational efficiency Not suitable for towing owing to the characteristics of speed Complicated launching and recovery operations High costs of production (hull characteristics that can fold and unfold the wings of a ship)
Other craft types	More effective for a particular task in a particular hull. Used to overcome the limitations of space, movement, and temperature that humans cannot tolerate.

**Table 2:** Classification and mission of the US Navy standard USV [47].

Class	Description
X-class (small, ~3 m)	Special operation forces (SOF) support Depends on the missions’ inexpensive consumables, specially built and served with unimportant details (high-level sonar and lidar, etc.) Not standardized and made for specific purposes Maritime interdiction operation missions ‘Low-end’ ISR L and B from 11 M rigid inflatable boat or combat rubber raiding craft
Harbor class (7 m)	Size of a boat on most naval vessels used for maritime security Performs most basic tasks ISR/gun payloads Mine countermeasure (MCM) delivery Surface warfare (SUW) SOF support
Snorkeler class (7 m SS)	Works more reliably than do other USVs on the high seas MCM search ASW (maritime shield) Special missions support (stealthy profile)
Fleet class (11 m)	Planar or semiplanar hull Durable because of usage MCM sweep Maritime shield SUW, gun and torpedo “High-end” SOF High power electronic warfare

**Table 3:** Classification of UUVs by weight [1].

Type	Weight (kg)	Diameter (cms)	Comments
Small UUV or man-portable vehicle class	11~45	>7.5 and <25	Inexpensive, economical, small turning radius, and maneuverable Increased work efficiency for specialized applications in various environments [72] Bionic AUV imitates the shape of the fish (hydrologically characterized by easy acceleration with less muscle and effort in the water) [73] and maintains the characteristics of mini/micro AUVs Riptide, Charlie, RoboPike, Ariel II, RoboLobster (lobster), and MT1 (fish robot) [74]
Mini-AUV	20~100		
Micro-AUV	<20	21.5	
Medium UUV or lightweight vehicle class	225	>25 and <53	Surveillance reconnaissance, mine removal, special-purpose marine investigation, network attack, and mobile communication node provision.
Large UUV or Heavyweight vehicle class	1350	>53 and <210	Continuous tactical surveillance and reconnaissance Covert reconnaissance Submarine deception Coastal access-based maritime surveys
Extra-large UUV or large vehicle class	10,150	>210	Continuous surveillance and reconnaissance, ambush-type ASW, long-distance maritime investigation, and transport for special operations

**Table 5:** Mass levels of control according to the IMO in the frame of a regulatory scoping exercise from 2018 [121].

Level	Description
1	Ships with automated processes and decision support
2	Remotely controlled ships with seafarers onboard
3	Remotely controlled ships without seafarers on board
4	Fully autonomous ships

**Table 6:** Single intelligence algorithms (UUVs).

Problem	Resolution	Performance and Additional Explanation	Ref.
UUV			
Route planning	Alarm pheromone-assisted ant colony system (AP-ACS)	Improve the robustness of the algorithm Better suited for route planning within complex real-world underwater environments Underwater environment models consider both seamounts and suspended objects All algorithms are coded in C++, and results are visualized in MATLAB 2017	[128]
AUV failure detection and control	Intelligent decision-making (IDM)	IDM and a fuzzy expert system (FES) System is fast and functions in real time Used for recognition and detection Route is determined via calculation every 20 nanoseconds with a 50 MHz clock.	[129]
Route planning	Improved bio-inspired neural network	Improved bio-inspired neural network Short and smooth route planning possible Can handle real-time route planning issues Target attractor concept + ANN	[130]
Route planning	3D cubic Bezier curve method	3D cubic Bezier curve method Enables the AUV to determine the shortest path with good continuity Can solve the problem of large distances between Bezier curves and the last number of objects	[131]

**Table 7:** Single intelligence algorithms (USVs).

Problem	Resolution	Performance and Additional Explanation	Ref.
USV			
Local obstacles avoiding	LROABRA (local reactive obstacle avoidance based on region analysis)	Radar, binocular vision, stereo vision, monocular vision, infrared cameras, and laser range finders are used. Stability of LROABRA is better than that of OAABHW High-speed (≥20 knots) USVs	[132]
Fast long-distance ship route planning	Multiscale visibility graph (VG) method	The number of visibility points can be reduced by half, and the VG search time can be shortened The local planning window (LPW) plays a role in greatly reducing the complexity of the VG model. Plan routes by simplifying the map using convex points of the obstacle polygon	[133]
Obstacle avoidance	Improved VFH algorithm	Partial encounter geometry model also used. Achieving collision avoidance in compliance with the international regulation COLREGSPerforming collision avoidance measures in a water environment with sudden and dynamic obstacles. Uses the CRI values of the obstacles as key parameters in the histogram and removing the grid model to speed up calculations and improve thresholds	[134]
Obstacle avoidance	Improved ant colony optimization (IACO) algorithm	Risk avoidance from steering during high-speed navigation in real and dynamic environments Implement and simulate static unknown environments and dynamic known environments (convergence, real-time performance, and stability of the improved ACO) in the cross-platform framework.	[135]
Obstacle avoidance	Genetic collision avoidance algorithm	Search ability, convergence speed, and local optimum are improved compared to ACO. Can effectively avoid multiple obstacles coming from different directions and conditions DCPA (distance of closest point of approach), TCPA (time of closest point of approach) are used. Simulation data such as the distance between the ASV and the obstacle vessel indicate that the collision avoidance behavior is safe and verify the feasibility of the proposed genetic collision avoidance algorithm.	[136]
Obstacle avoidance	Fuzzy inference algorithm	Long-range lidar, radar, and camera-based tracking technologies are used. Effective autonomous navigation and anticollision capacity Aragon USV (8 m) Calculation of fuzzy inference algorithm using TCPA and DCPA	[137]

**Table 8:** Main Projects to develop autonomous vessels [138,139].

Project Name	Participating Institutions
MUNIN (2012~2015)	8 EU research and industry
ReVolt (2014~2018)	DNV GL, NTNU
AAWA (2015~2018)	Rolls Royce, DNV GL, etc.
YARA BIRKELAND (2017~2020)	KM, YARA, NTUN, DNV GL
AUTOSHIP (2019~2022)	CIAOTECH, KM, SINTEF, BV

**Table 9:** Multi robots and swarm intelligence algorithms (USVs).

Problem	Resolution	Performance and Additional Explanation	Ref.
USV			
Underwater cooperative navigation techniques	SFE algorithms	Navigate with frame providing spatial density of plastics over sea. Differential evolution algorithm for control SFE algorithm is better suited for plastic collection than is ACO Development of SFE algorithm based on stigmergy and flocking for marine plastic collection	[166]
Obstacle avoidance	Combining restricted A* algorithms	Path planning by a constrained A* algorithm leader–follower formation control Maneuverability that allows for improved path-following performance for navigation and reduction of cross-track errors All followers are affected by the leader and all other USVs in the group, which is also applicable to UAVs Combining a limited A* algorithm using an artificial potential field based on USV various maneuvering response time capabilities	[167]
Obstacle avoidance	APF-DQN (artificial potential field-deep Q-learning network)	N: local dynamic path planning G: APF-DQN C: Markov decision process Performance of DRL-based method works better on the global trajectory A deep reinforcement learning and artificial potential field (APF)-based path planning method that complies with the International Regulations for Preventing Collisions at Sea (COLREGS) rules. Improvement of action space and reward function of a deep Q-learning network (DQN) by utilizing the APF method Eliminate USV with known local dynamic environment information Solve collision path planning challenge	[168]

**Table 10:** Multi robot and swarm intelligence algorithms (UUVs).

**Problem**	**Resolution**	**Performance and Additional Explanation**	**Ref.**
**UUV**			
Multi-AUV cooperation method	End-to-end MARL (multiagent reinforcement learning)	Markov decision process for navigating. CT-DE (centralized training with distributed execution) for path planning Obtain data through equipped sonars, electronic compasses, and inertial sensors via the Markov decision process MADDPG (multiagent deep deterministic policy gradient) algorithm is used for the end-to-end AUV control algorithm	[169]
Multi-AUV cooperation method	Genetic algorithm	Possible cost-performance trade-off Simulate up to 3 AUVs Automatically recharge energy at stationary charging stations The trajectories and positions of the AUV and charger are generated after utilizing the genetic algorithm as a global optimization too	[170]
Multi-AUV cooperation method and obstacle avoidance	Bio-inspired neural network algorithm	Bio-inspired neural network algorithm is used for path planning Shorter length of the trajectories than that of the artificial potential field method A 3D grid-based active model expressed as a bio-inspired neural network algorithm Simulation is conducted with conditions such as the presence of obstacles and different densities of obstacles	[171]
Multi-AUV cooperation method and network architecture	Underwater cooperative navigation technique based on SDN	Adaptive optimization policy for C-AUVs and predefined fixed spiral elliptic trajectory from top to bottom for S-AUVs are sued. Centralized network management Good performance in terms of execution efficiency and system stability Easier to deploy and more efficient in planning the AUV’s cruising trajectory	[172]
Route planning	Hybrid path planning	Shorten algorithm execution time and elimination of nonexecutable paths Detect obstacles using multibeam forward-seeking sonar (FLS) and create outlines (polygons) of obstacles Hybrid path planning algorithm based on PSO and waypoint guidance	[173]
Route planning	SAC (soft actor–critic) algorithm	dynamic detection scheme is used for path planning C: SDN (Software-Defined Networking) controller underwater diffusion source route planning for Pollution Detection Leading the Paradigm of Multi-AUV Network Intelligent Transportation Systems (SDNA-ITS)	[174]

**Table 11:** Heterogeneous cooperation intelligence algorithms.

Problem	Resolution	Performance and Additional Explanation	Ref.
Heterogeneous system formation (UAV–USV–UUV)	DQN (deep Q-learning) algorithm	LoS (line of sight) (UUV–USV) and underwater acoustic channel (USV–UUV) Markov decision process for control A success rate of target hunting over 95% A joint 3U heterogeneous system Balanced system energy consumption and interconnectivity	[180]
USV–UAV Systems	Multiultrasonic joint dynamic positioning algorithm	Multiultrasonic joint dynamic positioning algorithm G: hierarchical landing guide point generation algorithm and cubic B-spline curves UAV can land on the USV in 10 min The multiultrasonic joint dynamic positioning algorithm is based on ToA, which shows the position of the UAV in real time Cooperation mechanism and motion environment research	[181]
USV–UAV structure	CamShift algorithm and Douglas–Peucker algorithm	Turning mode and PID mode for control Useful for real-life maritime search and lifesaving missions Rescue operation using USV–UAV cooperation Cover and recognize a wider area by inspecting the scene with a UAV USVs bring people to shore, act as buoys, and distribute life jackets.	[182]
UAV–USV–AUV path planning	IPSO (improved particle swarm optimization) algorithm	UAV–USV–AUV systems are more efficient than are USV–AUV systems in performing search and tracking (SAT) missions Study of cooperative path planning problem for search and tracking (SAT) missions for underwater targets using UAV–USV–AUV cooperation The motion of a vehicle is expressed by the equations of motion	[183]

## Data Availability

Not applicable.
